# Polysomnography in the evaluation of traumatic brain injury

**Published:** 2012-11

**Authors:** Mohammad Rasoul Ghadami

**Affiliations:** ^a^Sleep Research Center, Kermanshah University of Medical Science, Kermanshah, Iran.

## Abstract

**Background:**

Traumatic brain injury (TBI) is a common problem and leading causes of morbidity and mortality in the general population. Sleep disorders are a common finding after the acute and chronic phase of TBI. They result in daytime somnolence which in turn may lead to poor daytime performance, altered sleep-wake schedule, heightened anxiety, and poor individual sense of well-being, insomnia and depression. Studies underscore the importance of examining the architecture of sleep in TBI patients that can use as objective diagnostic or prognostic markers of injury. Posttraumatic hypersomnia, sleep apnea, narcolepsy, periodic limb movement disorder (PLMD), Insomnia and Parasomnia because of REM behavior disorder (RBD) are the most common sleep disorders in TBI patients.

The neuropathology associated with TBI will depend on the nature and location of the underlying injury. Sleep polysomnography (PSG) analyses may provide a somewhat crude biomarker of injury as an initial step in the diagnostic work-up. If abnormalities in the PSG are observed, more detailed electroencephalographic methods, using electrodes at multiple locations (frontal, temporal, occipital) could be further used to localize the site of the most severe lesions.

**Conclusions:**

Additional research will be required to determine whether the location and severity of sleep PSG abnormalities can be used as a predictor for longer-term disability. The present study suggest that sleep measures may be a sensitive measure of brain injury after TBI and, in theory, could be used for determining the anatomy of brain injury.

**Keywords:**

Traumatic brain injuries, Polysomnography, Sleep disorders


					Volume 4, Suppl. 1 Nov 2012
Publisher: Kermanshah University of Medical Sciences
URL: http://www.jivresearch.org
Copyright:
Congress of Iranian Neurosurgeons, 14-16 November 2012, Kermanshah, Iran
Paper No. 45
Polysomnography in the evaluation of traumatic brain injury
Mohammad Rasoul Ghadami a,*
a
Sleep Research Center, Kermanshah University of Medical Science, Kermanshah, Iran.
Abstract:
Background: Traumatic brain injury (TBI) is a common problem and leading causes of morbidity and mortality in the
general population. Sleep disorders are a common finding after the acute and chronic phase of TBI. They result in
daytime somnolence which in turn may lead to poor daytime performance, altered sleep-wake schedule, heightened
anxiety, and poor individual sense of well-being, insomnia and depression. Studies underscore the importance of
examining the architecture of sleep in TBI patients that can use as objective diagnostic or prognostic markers of
injury. Posttraumatic hypersomnia, sleep apnea, narcolepsy, periodic limb movement disorder (PLMD), Insomnia and
Parasomnia because of REM behavior disorder (RBD) are the most common sleep disorders in TBI patients.
The neuropathology associated with TBI will depend on the nature and location of the underlying injury. Sleep
polysomnography (PSG) analyses may provide a somewhat crude biomarker of injury as an initial step in the
diagnostic work-up. If abnormalities in the PSG are observed, more detailed electroencephalographic methods, using
electrodes at multiple locations (frontal, temporal, occipital) could be further used to localize the site of the most
severe lesions.
Conclusions: Additional research will be required to determine whether the location and severity of sleep PSG
abnormalities can be used as a predictor for longer-term disability. The present study suggest that sleep measures
may be a sensitive measure of brain injury after TBI and, in theory, could be used for determining the anatomy of
brain injury.
Keywords:
Traumatic brain injuries, Polysomnography, Sleep disorders
* Corresponding Author at:
Mohammad Rasoul Ghadami: Sleep Research Center, Kermanshah University of Medical Science, Kermanshah, Iran, Cell phone: +989188332841
Email: mr_ghadami@yahoo.com, (Ghadami MR.).

				

